# How to treat combined respiratory and metabolic acidosis after extracorporeal cardiopulmonary resuscitation?

**DOI:** 10.1186/s13054-019-2461-2

**Published:** 2019-05-21

**Authors:** Xavier Bemtgen, Florentine Schroth, Tobias Wengenmayer, Paul M. Biever, Daniel Duerschmied, Christoph Benk, Christoph Bode, Dawid L. Staudacher

**Affiliations:** 1 Department of Cardiology and Angiology I, Heart Center Freiburg University, Hugstetter Straße 55, Freiburg, 79106 Germany; 2 Department of Cardiovascular Surgery, Heart Center Freiburg University, Hugstetter Straße 55, Freiburg, 79106 Germany

**Keywords:** VA-ECMO, eCPR, Survival, Carbon dioxide, paCO_2_, pH

Establishing a venoarterial extracorporeal membrane oxygenation (vaECMO) in cardiac arrest is known as extracorporeal cardiopulmonary resuscitation (eCPR). After eCPR, patients commonly present with a combined respiratory and metabolic acidosis [1]. It is clear that acidosis negatively impacts survival after eCPR [2] and that a respiratory acidosis can be easily corrected by vaECMO. Current guidelines for conventional CPR suggest normocapnia as targeted after return of spontaneous circulation [3]. This recommendation is based on heterogeneous data. While a recent meta-analysis found adverse outcome in both hyper- and hypocapnia [4], a randomized trial reported no difference in survival in low normal and high normal paCO_2_ [5].

The aim of the present study was to correlate arterial paCO_2_ and pH with hospital survival in eCPR.

A single-center retrospective register analysis was performed. All eCPR patients treated between 2010 and 2017 were included. We analyzed arterial blood gases after 1 h, 3 h, 6 h, 12 h, and 24 h as well as hospital mortality. We detected a total of 186 eCPR. The mean age was 58.6 ± 14.9 years, and total hospital survival rate was 26.3%. After cannulation, paCO_2_ and pH values were (mean ± standard deviation) 38.3 ± 8.9 mmHg/7.28 ± 0.14 (+ 1 h), 38.5 ± 8.5 mmHg/7.30 ± 0.11 (+ 3 h), 38.72 ± 7.42 mmHg/7.31 ± 0.11 (+ 6 h), 38.62 ± 7.26 mmHg/7.34 ± 0.10 (+ 12 h), and 38.22 ± 5.62 mmHg/7.38 ± 0.09 (+ 24 h), respectively. When comparing patients with paCO_2_ < 35, 35–45, and > 45 mmHg, survival was statistically similar for all observed time points. There was however a highly significant association between hospital survival and pH when comparing groups with pH < 7.3, 7.3–7.4, and > 7.4 (see Fig. 1).

**Fig. 1:**
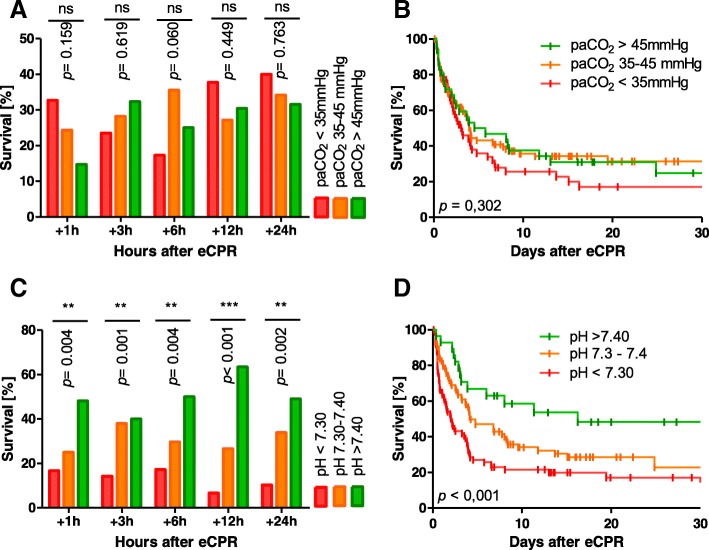
Survival of eCPR patients according to pH and paCO_2_ values at different time points. No significant correlation of survival and paCO_2_ level could be detected at any analyzed time point whereas pH was highly correlated with survival (**a**, **c**, chi-square shown in graph). **b**, **d** Example Kaplan-Meier survival curves according to paCO_2_ and pH 6 h after eCPR (log-rank tests shown in graph)

As secondary endpoint and surrogate for neurological outcome, neuron-specific enolase (NSE) was analyzed. Maximum NSE measured within 72 h after eCPR was 150.8 ± 145.1 μg/l (mean ± standard deviation). When correlating maximum NSE with paCO_2_ at 1, 3, 6, 12, and 24 h after eCPR, no statistical significant linear correlation was found (*p* > 0.4 for all time points). There was however a significant linear correlation of maximum NSE and pH at 1, 3, and 6 h after eCPR (*p* = 0.037, 0.029, and 0.018, respectively).

In this registry study, we found a strong correlation between hospital survival and arterial pH but no such correlation with paCO_2_. Also elevated NSE as a marker for neural injury did correlate with pH but not with paCO_2_. Being a retrospective, observational, single-center study, inherent limitations and biases are to be presumed and findings are to be considered hypothesis generating. Until further data are available however, it might be reasonable to correct both respiratory and metabolic acidosis in eCPR patients.

## Abbreviations

eCPR: Extracorporeal cardiopulmonary resuscitation
NSE: Neuron-specific enolase
paCO_2_: Partial pressure of carbon dioxide in arterial blood
vaECMO: Venoarterial extracorporeal membrane oxygenation
